# 

**Published:** 1903-12

**Authors:** 


### BOOKS RECEIVED.

The American Illustrated Medical Dictionary. For Practitioners and Students. A Complete Dictionary of the Terms used in Medicine, Surgery, Dentistry, Pharmacy, Chemistry, and the kindred branches, including much collateral information of an encyclopedic character, together with new and elaborate tables of Arteries, Muscles, Nerves, Veins, and the like; of Bacilli, Bacteria, Micrococci, Streptococci; Eponymic Tables of Diseases, Operations, Signs and Symptoms, Stains, Tests, Methods of Treatment, etc., etc. By W. A. Newman Dorland, A.M., M.D., editor of the "American Pocket Medical Dictionary." Handsome large octavo, nearly 800 pages, bound in full flexible leather. Philadelphia, New York, London: W. B. Saunders & Company. 1903. (Price, $4.50 net; with thumb index, $5.00 net.)

A Textbook of Clinical Anatomy. For Students and Practitioners. By Daniel N. Eisendrath, A.B., M.D., Clinical Professor of Anatomy in the Medical Department of the University of Illinois (College of Physicians and Surgeons); Attending Surgeon to the Cook County Hospital, Chicago. Handsome octavo of 515 pages, with 153 illustrations, a number in colors. Philadelphia, New York, London: W. B. Saunders & Company. 1903. (Cloth, $5.00 net; sheep or half morocco, $6.00 net.)

A Textbook upon the Pathogenic Bacteria. For Students of Medicine and Physicians. By Joseph McFarland, M.D., Professor of Pathology and Bacteriology in the Medico-Chirurgical College, Philadelphia; Pathologist to the Philadelphia Hospital and to the Medico-Chirurgical Hospital, Philadelphia. Handsome octavo volume of 629 pages, fully illustrated, a number in colors. Philadelphia, New York, London: W. B. Saunders & Company. 1903. (Cloth, $3.50 net.)

A Textbook of Diseases of Women. By Barton Cooke Hirst, M.D., Professor of Obstetrics in the University of Pennsylvania; Gynecologist to the Howard, the Orthopedic, and the Philadelphia Hospitals. Handsome octavo volume of 675 pages, with 650 mostly original illustrations, many in colors. Philadelphia, New York, London: W. B. Saunders & Co. 1903. (Cloth, $5.00 net; sheep or half morocco, $6.00 net.)

General Pathology, or the Science of the Causes, Nature and Course of the Processes of Disease. By Dr. Ernest Ziegler, Professor of Pathological Anatomy and of General Pathology in the University of Freiburg in Breisgau. Translated from the tenth revised German edition, and edited by Alfred Scott Warthin, M.D., Professor of Pathology and Director of the Pathological Laboratory in the University of Michigan, Ann Arbor, Mich. Octavo, pp. 783. Illustrated. New York: William Wood & Co. 1903. (Cloth, $5.00.)

A Textbook on the Practice of Medicine. Designed for the Use of Students. By James Magoffin French, M.D., Lecturer on the Theory and Practice of Medicine, Medical College of Ohio; Attending Physician, St. Mary's Hospital, Cincinnati. Octavo, pp. 799. Illustrated by ten full-page plates and fifty wood engravings. New York: William Wood & Company. 1903. (Cloth, $4.00.)

International Clinics. A Quarterly of Illustrated Clinical Lectures and especially prepared original articles on Treatment, Medicine, Surgery, Neurology, Pediatrics, Obstetrics, Gynecology, Orthopedics, Pathology, Dermatology, Ophthalmology, Otology, Rhinology, Laryngology, Hygiene and other topics of interest to students and practitioners, by leading members of the medical profession throughont the world. Edited by A. O. J. Kelly, A.M., M.D., Philadelphia. Volume III. Thirteenth series. 1903. Philadelphia: J. B. Lippincott Company. 1903. [Cloth, $2.00.]

Electro-Static Modes of Application, Therapeutics and the Uses of the Röntgen Ray. By William Benham Snow, M.D., Professor of Electro-Therapeutics and Radiotherapy in the New York School of Physical Therapeutics. Second edition revised and enlarged. Over one hundred illustrations, including ten full page half-tones. A. L. Chatterton & Co. New York. 1903. (Price, $3.00.)

Transactions of the Medical Association of the State of Alabama. Annual meeting held at Talladega, Ala., April 21-24, 1903. G. P. Waller, M.D., Secretary.

Annual Report of the Supervising Surgeon General of the Marine Hospital Service of the United States for the fiscal year 1900. Two volumes. Washington: Government Printing Company. 1903.

Transactions of the American Otological Society. Thirty-sixth annual meeting held at Washington, D. C., May 12 and 13, 1903. Volume VIII., Part II. F. L. Jack, M.D., Secretary.

Lea's Medical Epitome Series. Physics and Inorganic Chemistry. A Manual for Students and Practitioners. By A. McGlannan, M.D., Associate Professor of Physiological Chemistry, College of Physicians and Surgeons, Baltimore, Md. Duodecimo, pp. 216. Illustrated with 20 engravings. Series edited by V. C. Pedersen, A.M., M.D., Instructor in Surgery at the New York Polyclinic Medical School and Hospital. Lea Brothers & Co., Philadelphia and New York. 1903. (Price, $1.00.)

The Four Epochs of Woman's Life. Maidenhood, Marriage, Maternity, Menopause. By Anna M. Galbraith, M. D., Author of Hygiene and Physical Culture for Women. With an introductory note by John H. Musser, M.D., Professor of Clinical Medicine, University of Pennsylvania, Duodecimo volume of 247 pages. Second edition, revised and greatly enlarged. Philadelphia, New York, London: W. B. Saunders & Company. 1903. (Cloth, $1.50 net.)

The Johns Hopkins Hospital Reports. Volume XI. Nos. 1-9. Baltimore: The Johns Hopkins Press. 1903.

Medical Reports of the Sheppard and Enoch Pratt Hospital. Volume I. No. 1. Baltimore: The Friedenwald Co. 1903.

A Manual of Bacteriology. By Herbert U. Williams, M. D., Professor of Pathology and Bacteriology, Medical Department, University of Buffalo. Duodecimo, pp. 351. With 99 illustrations. Third edition, revised and enlarged. Philadelphia: P. Blakiston's Son & Co. 1903. (Price, $1.75.)

Clinical Treatises on the Pathology and Therapy of Disorders of Metabolism and Nutrition. By Prof. Dr. Carl von Noorden, Physician-in-Chief to the City Hospital, Frankfurt a. M. Authorised American edition, translated under the direction of Boardman Reed, M. D., Professor of Diseases of the Gastrointestinal Tract, Hygiene and Climatology, Department of Medicine, Temple College; Physician to the Samaritan Hospital, Philadelphia. Part IV. The Acid Autointoxications. By Prof. Dr. Carl von Noorden and Dr. Mohr. Duodecimo, pp. 80. New York: E. B. Treat & Co. 1903. (Price, 50 cents.)

Lea's Series of Medical Epitomes. Normal Histology. A Manual for Students and Practitioners. By John R. Wathen, M. D., Professor of Surgery and Gynecology in the Kentucky School of Medicine, Louisville. Series edited by V. C. Pedersen, M. D., Instructor in Surgery at the New York Polyclinic Medical School and Hospital. Duodecimo, pp. 229. Illustrated. Lea Brothers & Co., Philadelphia and New York. 1903. (Price, $1.00.)

Blakiston's Quiz Compends. Compend of Gynecology. By William H. Wells, M. D., Chief of the Gynecological Staff of the Mount Sinai Hospital, Philadelphia. Third edition, revised and enlarged. With 145 illustrations. Philadelphia: P. Blakiston's Son & Co. 1903. (Price, 80 cents.)

A Textbook of Practical Gynecology for Practitioners and Students. By D. Tod Gilliam, M. D., Professor of Gynecology in Starling Medical College, Columbus, O.; Gynecologist to St. Anthony and St. Francis Hospitals, Columbus, O. Octavo, pages 650. Illustrated with 350 engravings, a colored frontispiece and full page half-tone plates. Philadelphia: F. A. Davis Company. 1903. (Cloth, $4.00 net; half-Russia, $5.00 net, delivered.)

Lea's Series of Medical Epitomes. Anatomy. A Manual for Students and Physicians. By Henry E. Hale, A. M., M. D., Assistant Demonstrator of Anatomy, College of Physicians and Surgeons (Columbia University) New York. Duodecimo, 384 pages, with 71 illustrations. Series edited by V. C. Pedersen, M. D., Instructor in Surgery at the New York Polyclinic Medical School and Hospital. Lea Brothers & Co., Philadelphia and New York. 1903. (Cloth, $1.00 net.)

Infectious Diseases. Their Etiology, Diagnosis and Treatment by G. H. Roger, Professor Extraordinary in the Faculty of Medicine of Paris, etc., translated by M. S. Gabriel, M. D., New York. In one octavo volume, of 864 pages, with 43 illustrations. Lea Brothers & Co., Philadelphia and New York. 1903. (Cloth, $5.75 net.)

Transactions of the twenty-fifth annual meeting of the American Laryngological Association, held at Washington, D. C., May 12-14, 1903. James E. Newcomb, M.D., Secretary. New York: Rooney-Otten Co.

The Treatment of Certain Malignant Growths by Excision of the External Carotids. By Robert H. M. Dawbarn, M. D., Professor of Surgery and Surgical Anatomy in the New York Polyclinic Medical School and Hospital; Visiting Surgeon to the City Hospital, New York. (The Samuel D. Gross Prize Essay). Octavo, pages 105. Philadelphia: F. A. Davis Co. 1903. (Price, $2.00 net.)

A Treatise on Orthopedic Surgery. By Royal Whitman, M. D., Instructor in Orthopedic Surgery in the College of Physicians and Surgeons (Columbia University), New York. Second edition, revised and enlarged. Octavo, 820 pages, with 507 engravings, mostly original. Lea Brothers & Co., Philadelphia and New York. 1903. (Cloth, $5.50 net.)

Clinical Pathology of the Blood. A Treatise on the General Principles and Applications of Hematology. By James Ewing, M. D., Professor of Pathology, Cornell University Medical College, New York. Second edition, revised and enlarged. Octavo, 492 pages, with 43 engravings and 18 full page colored plates. Lea Brothers & Co., Philadelphia and New York. 1903. (Cloth, $3.50, net.)

A Manual of Hygiene and Sanitation. By Seneca Egbert, M. D., Professor of Hygiene in the Medico-Chirurgical College of Philadelphia. Third edition, enlarged and thoroughly revised. Duodecimo, 467 pages, with 86 illustrations. Lea Brothers & Co., Philadelphia and New York. 1903. (Cloth, $2.25 net.)

Atlas of the External Diseases of the Eye. By Prof. Dr. O. Haab, of Zurich. Second edition, thoroughly revised. Edited, with additions, by G. E. de Schweinitz, A. M., M. D., Professor of Ophthalmology in the University of Pennsylvania. With 98 colored lithographic illustrations on 48 plates, and 232 pages of text. Philadelphia, New York, London: W. B. Saunders & Company. 1903. (Price, $3.00 net.)

The American Pocket Medical Dictionary. Edited by W. A. Newman Dorland, M. D., Assistant Obstetrician to the Hospital of the University of Pennsylvania. Containing the pronunciation and definition of the principal words used in medicine and kindred sciences, with 566 pages and 64 extensive tables. Philadelphia, New York, London: W. B. Saunders & Company. 1903. (Flexible leather, with gold edges, $1.00 net; with thumb index, $1.25 net.)

Report of the Commissioner of Education for the year 1902. Volume I. Washington: Government Printing Office. 1903.

